# MicroRNAs Modulate the Pathogenesis of Alzheimer’s Disease: An In Silico Analysis in the Human Brain

**DOI:** 10.3390/genes11090983

**Published:** 2020-08-24

**Authors:** Agnese Gugliandolo, Luigi Chiricosta, Virginia Boccardi, Patrizia Mecocci, Placido Bramanti, Emanuela Mazzon

**Affiliations:** 1 IRCCS Centro Neurolesi “Bonino Pulejo”, Via Provinciale Palermo, Contrada Casazza, 98124 Messina, Italy; agnese.gugliandolo@irccsme.it (A.G.); luigi.chiricosta@irccsme.it (L.C.); placido.bramanti@irccsme.it (P.B.); 2 Department of Medicine, Santa Maria della Misericordia Hospital, Section of Gerontology and Geriatrics, University of Perugia, Piazzale Gambuli 1, 06132 Perugia, Italy; virginia.boccardi@unipg.it (V.B.); patrizia.mecocci@unipg.it (P.M.)

**Keywords:** Alzheimer’s disease, microRNA, in silico analysis, human brain, amyloid β

## Abstract

MicroRNAs (miRNAs) are small RNAs involved in the post-transcriptional regulation of their target genes, causing a decrease in protein translation from the mRNA. Different miRNAs are found in the nervous system, where they are involved in its physiological functions, but altered miRNAs expression was also reported in neurodegenerative disorders, including Alzheimer’s disease (AD). AD is characterized by memory loss, cognitive function abnormalities, and various neuropsychiatric disturbances. AD hallmarks are amyloid β (Aβ) aggregates, called senile plaques, and neurofibrillary tangles (NFTs) formed by hyperphosphorylated Tau protein. In this study, we performed an in silico analysis to evaluate altered patterns of miRNAs expression in the brains of AD patients compared to healthy subjects. We found 12 miRNAs that were differentially expressed in AD compared to healthy individuals. These miRNAs have target genes involved in AD pathogenesis. In particular, some miRNAs influence Aβ production, having as target secretase and amyloid precursor protein (APP). Some miRNAs were reported to be involved in nervous system functions, and their alteration can cause neuronal dysfunction.

## 1. Introduction

MicroRNAs (miRNAs) are small RNAs, 21–23 nucleotides in length, that mediate the post-transcriptional regulation of their target genes, causing a reduction of protein translation from the mRNA. In particular, miRNAs cause translational inhibition and the promotion of mRNA decay [1]. miRNAs have been found in the nervous system, where they may regulate physiological processes such as neuronal differentiation and synaptic plasticity, but they may also play a role in pathological conditions, including neurodegenerative disorders such as Alzheimer’s disease (AD) [2].

AD represents the most common form of dementia, and the number of patients is arising due to the progressively increasing aged population. Clinically, AD is characterized by memory loss, damaged cognitive function, and other neuropsychiatric disturbances. The hallmarks of AD are senile plaques formed by the aggregated amyloid β (Aβ) peptide, and neurofibrillary tangles (NFTs) constituted by hyperphosphorylated Tau protein. 

**Figure 1:**
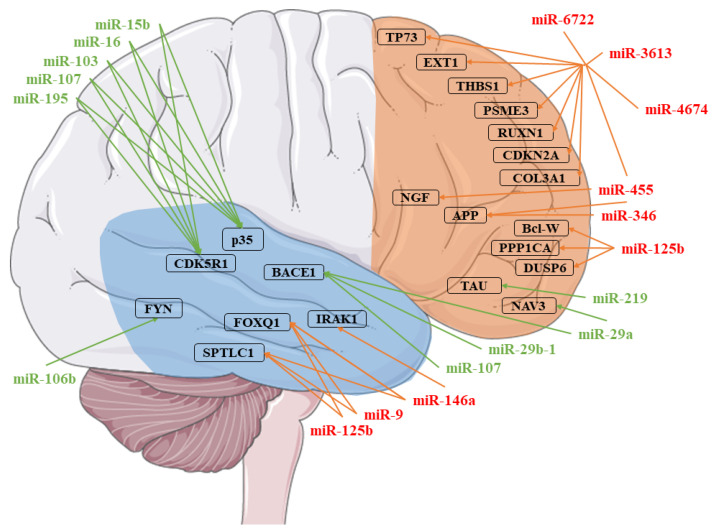
Representation of the microRNAs (miRNAs) involved in Alzheimer’s disease (AD). Each miRNA identified was associated with its targets. The miRNAs in red were found to be upregulated, while those in green are downregulated. The targets are identified in the temporal (blue) or the frontal (orange) cortex [5,6,7,8,9,10,11,12,13,14,15,16]. The figure was drawn using the vector image bank of Servier Medical Art by Servier (http://smart.servier.com/). Licensed under a Creative Commons Attribution 3.0 Unported License (https://creativecommons.org/licenses/by/3.0/).

Aβ is produced from the cleavage of amyloid precursor protein (APP) by secretase. In particular, the amyloidogenic pathway involves the consecutive actions of two enzymes, β- and γ-secretases, leading to the production of Aβ [3]. Aβ_1–40_ is the most abundant form of Aβ in the brain, but Aβ_1–42_ is more toxic because of its tendency to aggregate and form oligomers. It is the Aβ isoform that is associated with AD [4]. On the contrary, in the non-amyloidogenic pathway, APP is cleaved by α- and γ-secretases [3]. Different studies have demonstrated the role of miRNAs in Aβ production and Tau hyperphosphorylation (Figure 1).

Indeed, the alterations of miRNAs can cause dysregulation in the pathways that mediate the balance between Aβ synthesis and clearance [17] or influence Tau phosphorylation through the activation of different kinases and phosphatases [18]. Several miRNAs, like miR-17, miR-106b, or miR-132, are able to bind the APP and the β-secretase 1 (BACE1) regulating the expression of Aβ [19]. In vitro and in vivo experiments have shown that the downregulation of miR-29a, miR29-b-1, mirR-29c, and miR-339-5p led to an increase in the levels of BACE1, and consequently, of Aβ [5,20,21]. The Tau expression is also directly regulated by miRNAs like miR-132 or miR-212, the deficiency of which can lead directly to the aggregation of phosphorylated Tau in mice [22]. Moreover, the overexpression of miR-125b results in a series of cascade of events that, via mitogen-activated protein kinase signaling, lead to Tau hyperphosphorylation [6]. 

New studies even suggest that the pathogenesis and progression of AD could be underpinned by miRNAs dysregulation [23]. Indeed, several experiments in animal models have shown that Aβ and Tau protein seems reduced in the brain after miRNAs regulation. For instance, the inhibition of miR-126, miR-128, and miR-125b leads to neuroprotective effects against Aβ_1–42_, improves cognitive capacity, and decreases Tau phosphorylation [24]. For this reason, miRNAs may be used to evaluate the stage and progression of AD [25].

The aim of this study was to discover miRNAs deregulated in the brain of AD patients, in order to clarify their role in the pathology. We carried out the study by analyzing the postmortem brain area obtained from Brodmann Area 9 (BA9) and Brodmann Areas 21/22 (BA21/22) of AD patients and healthy individuals. BA9 is the dorsolateral prefrontal cortex, and takes part in executive functions like planning, cognitive flexibility, and working memory [26]. BA21/22 are located in the temporal cortex and are involved in auditory and language processing, which shows the loss of the synapses in AD [27]. Thus, we analyzed the non-coding RNAs in order to find significantly up- and downregulated miRNAs in the brain of AD patients compared to controls. To obtain the data from the brain area we used the Sequence Read Archive (SRA) [28] repository, a collection of free available deposited data. We used the databases miRecords [29], miRTarBase [30], and tarBase [31], collecting predicted and validated genes targeted by miRNAs. In addition, we took advantage of the Kyoto Encyclopedia of Genes and Genomes (KEGG) pathway database [32], which provides a curated pathway of AD. 

## 2. Materials and Methods 

### 2.1. Sample Collection

The Gene Expression Omnibus (GEO) repository [33] was used to collect the human samples of the brains of AD patients and control individuals. In detail, the projects Gene Series Expression (GSE) GSE63501 [7] and GSE46131 [34] were used to enlarge our cohort. These two projects collect raw data obtained from healthy individuals and AD patients. Specifically, in the project GSE63501, only the BA9 was removed from postmortem brains, whereas only the BA21/22 was removed in the project GSE46131. 

**Figure 2:**
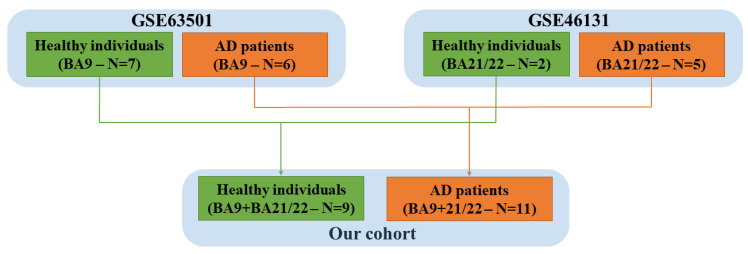
Our analysis was carried out on 9 healthy individuals (*n* = 7 GSE63501 and *n* = 2 GSE46131) and 11 Alzheimer’s disease (AD) patients (*n* = 6 GSE63501 and *n* = 5 GSE46131). GSE: Gene Series Expression.

As summarized in Figure 2, we put together the healthy individuals and the AD patients from the two different cohorts. Thus, in our study, we had 9 healthy individuals (control group) and 11 AD patients. In both cohorts, the non-coding RNAs were extracted from frozen brain tissue, and the raw data of the RNA sequences were obtained through high-throughput sequencing. 

### 2.2. miRNAs Selection

The raw data were obtained from the SRA repository in FASTQ format, and their quality was verified using the software FastQC. The low-quality bases and the adapters sequences were trimmed by Trimmomatic (Usadel Lab, Aachen, Germany) [35] (version 0.38). The reads were then aligned to the “miRNA” features obtained by the reference human genome GRCh38 using the Spliced Transcripts Alignment to a Reference (STAR) RNA-Seq aligner [36]. After sorting, the reads of the alignments were counted with the Python package htseq-count [37]. Finally, using DESeq2, in R, we computed the changes in the expression of miRNAs between healthy and AD subjects [38]. The *p*-value was corrected using the Benjamini–Hochberg procedure. No fold-change cutoff was used, but the genes with a *q*-value > 0.05 were rejected. Moreover, in order to associate each miRNA to its target gene, the package multiMiR of Bioconductor was used [39]. Specifically, the package was used to inspect all the validated target genes in miRecords, miRTarBase, and tarBase. Finally, the Bioconductor package KEGGREST was used to download and manipulate the map of the AD (hsa05010) from the KEGG database, while the package pathview [40] was used to depict the AD map with the genes targeted by our miRNAs.

## 3. Results

### RNA-Seq Analysis between Healthy Subjects and AD Patients

The RNA-Seq analysis between healthy individuals and AD patients revealed 12 miRNAs differentially expressed, associated with a statistical significance (Table 1). Specifically, *MIR199A2, MIR218-2, MIR24-2, MIR92A1,* and *MIR99A* were upregulated in AD, while *MIR129-2, MIR1296, MIR219A1, MIR29B1, MIR375, MIR411,* and *MIR431* were downregulated in AD. All the miRNAs had at least a one-fold increase or decrease. In detail, *MIR24-2* and *MIR99A* increased by 1-fold, while *MIR375, MIR411* and *MIR431* decreased by the same order. Two-fold deregulation was found for *MIR199A* and *MIR92A1* (increasing) and for *MIR129-2, MIR1296,* and *MIR29B1* (decreasing). *MIR218-2* increased by 3-fold, whereas *MIR219A1* decreased by 9-fold.

**Figure 3:**
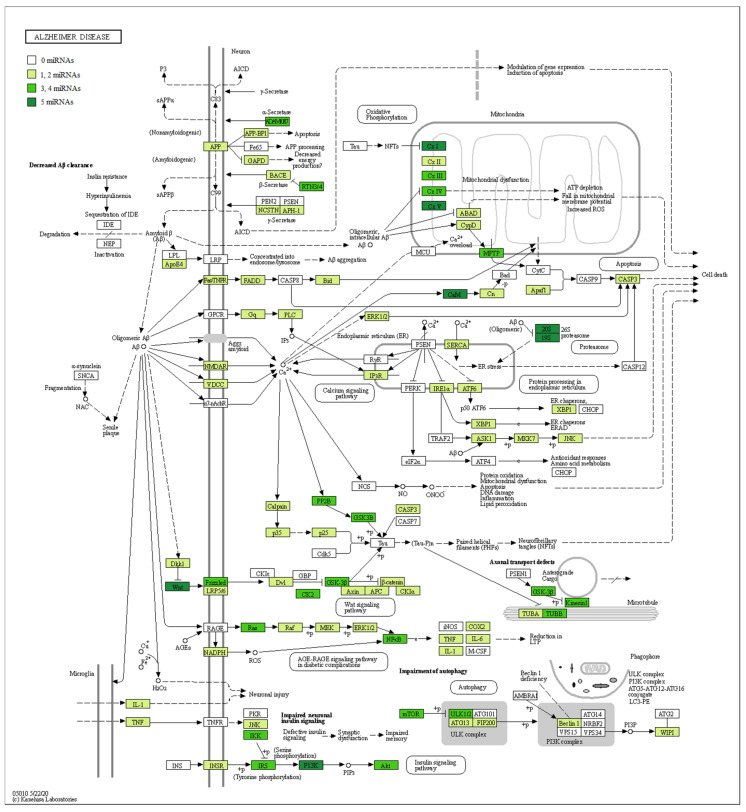
AD pathway with target genes of upregulated miRNAs highlighted. The schema represents the AD pathway in KEGG. The green scale represents the proteins encoded by genes known to be targets of the upregulated miRNAs. A darker color green, indicates a greater number of miRNAs that target the genes encoding that specific protein. If the protein is white, no miRNA targets any gene there. In particular, given that miRNAs are upregulated in our analysis, we can expect these genes to be downregulated.

**Figure 4:**
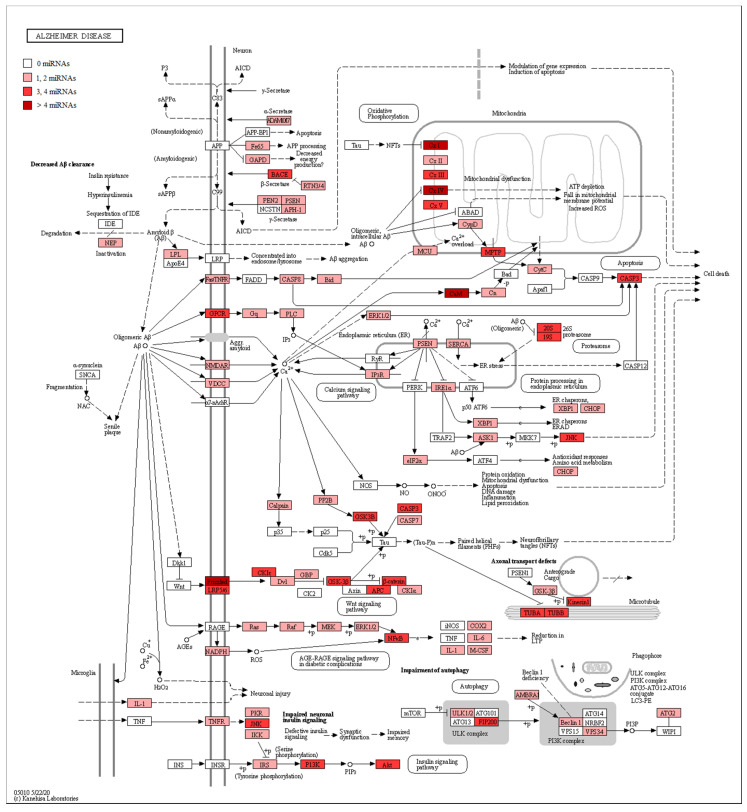
AD pathway with target genes of downregulated miRNAs highlighted. The schema represents the AD pathway in KEGG. The red scale represents the proteins encoded by genes known to be targets of downregulated miRNAs. A darker color, indicates a greater number of miRNAs that target the genes encoding that specific protein. If the protein is white, no miRNA targets any gene there. In particular, given that miRNAs are downregulated in our analysis, we can expect these genes to be upregulated.

In addition, in Table 2, we associated our miRNAs to their biological role in AD, their gene targets, and the pathway where they are involved. Furthermore, in order to evaluate the involvement of miRNAs in AD, we took advantage of the manually curated maps in the KEGG database. Specifically, the pathway hsa05010 highlights how Aβ is linked to pathological effects inside the neuron, altering the proteins’ signal transduction and leading to Aβ aggregation, mitochondrial dysfunction, and cell death. For this reason, we checked if the genes belonging to the AD pathway were reported as targets of our miRNAs. Our analysis evidenced that both the up- and downregulated miRNAs showed several target genes belonging to the AD pathway (Table S1). In detail, Figure 3 shows all the proteins targeted by upregulated miRNAs. Conversely, Figure 4 highlights the protein targets of our downregulated miRNAs. Interestingly, the genes involved in the mitochondrial complex are always highlighted. 

## 4. Discussion

miRNAs are gaining more and more attention as a mechanism to regulate gene expression. Abnormal levels of different miRNAs were found in different neurodegenerative disorders, including but not limited to AD [2,23,41], where they regulate pathways including axonal guidance, apoptosis, oxidative stress, and inflammation, indicating that these pathways are commonly altered features in neurodegeneration [42,43].

Regarding the hallmarks of AD, different miRNAs have been identified as involved in different aspects of AD pathogenesis, from APP expression and cleavage to Tau expression and NFT formation [23]. In terms of Aβ, different pathways are involved in its clearance, such as the ubiquitin-proteasome system, autophagy, degradation enzymes, and transport across the blood-brain barrier. Different miRNAs are known to modulate components of these pathways, influencing Aβ clearance [17].

As can be observed in Figure 1, many miRNAs have already been found deregulated in AD. Interestingly, preclinical studies of AD have already highlighted the dysregulation of several miRNAs in different cortex areas. For instance, miR-29a, miR-29b-1, and miR-107, which target the BACE1 protein, were found downregulated in the temporal cortex in two different studies [5,8]. Conversely, in the same cortex, miR-9, miR-125b, and miR-146a are upregulated, and target Serine Palmitoyltransferase Long Chain Base Subunit 1 (SPTLC1) and FOXQ1 [9]. In particular, miR-146a also targets IRAK1 [10]. miR-106b, miR-15b, miR-16, miR-103, miR-107, and miR-195 were also found to be downregulated in the temporal cortex. The first miRNA targets FYN [11], the other CDK5R1 and p35 [12]. Several miRNAs were also observed to be dysregulated in the frontal cortex. mir-29a is also downregulated in the frontal cortex, where it targets NAV3 [13]. miR-219 is also downregulated in the same cortex, which targets the Tau protein [7], while miR-125b is upregulated, which targets DUSP6, PPP1CA, and Bcl-W [6]. The miR-346 and miR-455 are upregulated, and they both target APP [15,16]. In addition, miR-455 also targets NGF, and along with miR-3613, miR-4674, miR6722, THBS1, COL3A1, RUXN1, TP73, EXT1, CDKN2A, and PSME3 [14,16]. Even if some of the miRNAs found in our analysis were already observed to be dysregulated in the cortex of AD patients, most of them had yet to be associated. These miRNAs were reported to regulate Aβ production and clearance. In particular, we found the downregulation of *MIR29B1*. A previous study demonstrated that miR29b1 could regulate *BACE1* expression in vitro [5]. Moreover, one miR-29b target is Sp1, which is involved in AD, leading to the production of *APP, BACE1*, and Tau. A significant increase of Sp1 expression, with a reduction of miR-29b, was observed in the peripheral blood mononuclear cells (PBMCs) of AD patients [44]. Interestingly, it was shown that the administration of exosomes containing miR-29b or the administration of pre-miR-29b might be promising therapeutic strategies against AD [45,46]. In addition, low miR-29b serum expression has been associated with a decreased thickness of bilateral temporal, right parietal, and right frontal regions [47]. miR-29b seems to also exert a positive action in neuron survival. Indeed, brain-specific knockdown of miR-29 caused cell death in the hippocampus and cerebellum. The neuronal cell death was associated with ataxia [48]. Then, *MIR29B1* downregulation may be involved in AD, both acting on genes directly involved in AD pathogenesis (e.g., *BACE1*), as well as acting indirectly, given its protective action on neuron survival.

Another miRNA that we found downregulated in AD is *MIR129-2*. Previously, miR-129-5p was found downregulated in AD and was associated with Aβ plaques and NFT [49]. The upregulation of miR-129-5p was shown to reduce nerve injury and inflammatory response in an experimental AD model, indicating that miR-129-5p may be a potential treatment against AD [50]. 

Additionally, *MIR219A1* was downregulated in our analysis, and it was already shown to be downregulated in the brain of AD patients. In particular, it was demonstrated in a *Drosophila* model that miR-219 reduction aggravated Tau toxicity. Indeed, miR-219 binds Tau mRNA, repressing its synthesis [7]. Moreover, miR-219 mitigated glutamate neurotoxicity in cultured hippocampal neurons, targeting calmodulin-dependent protein kinase II γ [51]. Another study found that miR-219-5p was downregulated in the brain of AD patients and associated with the increase of *TTBK1* and GSK-3β, influencing Tau phosphorylation [52]. 

Another miRNA upregulated in our analysis was *MIR99A*. In a previous study, another member of the family, miR-99b, was associated with NFT [49]. 

We also found the upregulation of *MIR24-2*. Nicastrin, a γ-secretase subunit, is a target of this miRNA, and with this mechanism, this miRNA seems able to reduce Aβ_42_ production in vitro [53]. However, miR-24 also targets *MMP14* [54], and it is known that MMP can degrade Aβ. Moreover, miR-24-3p was found to be deregulated in the plasma exosome and cerebrospinal fluid of AD patients [55,56].

Another upregulated miRNA found in the brain of AD patients was *MIR199A2*. A previous study demonstrated that miR-199a was involved in AD pathogenesis because it negatively regulated neuritin [57]. It is also involved in neurogenesis. In particular, miR-199 increased during early brain development and modulated extracellular signal-regulated kinase (ERK). The overexpression of miR-199 in wildtype mouse embryonic brains altered neurogenesis and neuronal migration [58]. However, miR-199a-3p can also impair the autophagic process [17]. 

We found a downregulation of *MIR375*. It was already demonstrated in a previous study [59] and a mouse model of tauopathy [60]. Moreover, it targets *UBE3A*, which is involved in AD pathogenesis [17,61].

Additionally, *MIR411* was downregulated in our analysis. It demonstrated neuroprotective effects in an acute spinal cord injury model, through the downregulation of FasL [62].

On the contrary, *MIR92A1* was upregulated in the brain of AD patients in our analysis. miR-92 was discovered to be involved in contextual fear memory. Among its targets are the genes *CPEB3, MEF2D,* and *KCC2*, involved in hippocampus-dependent memory and structural plasticity [63].

The Wnt/β-catenin signaling pathway exerts different roles in physiological brain function and in AD, preventing Aβ toxicity. It was shown that miR-431 exerted protective effects against Aβ toxic stimuli, silencing Krm1, that together with Dkk1 plays a role in the inhibition of the Wnt/β-catenin signaling pathway [64]. In the same way, it regulates axon regeneration and growth [65]. However, in our analysis, *MIR431* was downregulated, and thus it may not be able to protect against Aβ toxicity.

*MIR218-2* in our analysis was found to be upregulated. It was found that miR-218 is involved in the effect of the estrogen receptors on Tau phosphorylation. miR-218 expression was increased by the estrogen receptor α, and then miR-218 inhibited its target, the protein tyrosine phosphatase α (PTPα). PTPα reduction caused GSK3β activation and PP2A inactivation, which represent the main Tau kinase and phosphatase, respectively, inducing Tau phosphorylation [66]. Interestingly, *ADAM17*, encoding for an α-secretase, was discovered as a target of miR-218 in medulloblastoma. Additionally, genes encoding for components of the mitochondrial respiratory chains are miR-218 targets [67]. Thus, the upregulation of miR-218 may reduce the levels of α-secretase, decreasing the APP processing through the non-amyloidogenic pathway. Moreover, miR-218 modulated the Wnt pathway [68].

We observed the downregulation of miR1296. It is involved in the neuronal response to oxidative stress [69]. Moreover, it was found to be dysregulated in AD compared to patients with no cognitive impairment [70].

Several miRNAs have already been linked to AD, but many possible candidates are unknown. Our study shows that miRNAs associated with *MIR29B1, MIR129-2, MIR219A1, MIR199A2, MIR92A1,* and *MIR1296* were already observed in AD patients. On the other hand, *MIR99A, MIR24-2, MIR375, MIR411, MIR431,* and *MIR218-2* have not previously been studied or reported in the brain tissue of AD patients. Nevertheless, miR-24 was already detected in the cerebrospinal fluid of AD patients [59], while miR-375 was already reported in the plasma of a mouse model of AD [71]. In Table 2, we summarized the mechanisms regarding the miRNAs we found to be implicated in AD. For *MIR129-2*, *MIR1296*, and *MIR99A,* neither pathway nor target was identified. Moreover, the targets of *MIR199A2, MIR219A1, MIR24-2, MIR375, MIR411,* and *MIR92A1* are known, and their role in AD is not clear.

For this reason, we evaluated if the genes belonging to the AD pathway in KEGG were reported in the databases as targets of the miRNAs found to be differentially regulated. We observed that each miRNA targeted different genes belonging to this pathway, indicating their involvement in AD pathogenesis. In particular, Figure 3 and Figure 4 highlight the role of both the miRNAs up- and downregulated in the mitochondrial complexes. Moreover, the α-, β-, and some subunits of the γ-secretases are also targets of the miRNAs. This behavior can support the hypothesis of the key role of miRNAs in the pathogenesis of AD.

## 5. Conclusions

This analysis evidenced patterns of miRNAs extracted from postmortem brain samples differentially regulated in AD patients compared to healthy individuals. Our study showed that *MIR129-2, MIR1296, MIR219A1, MIR29B1, MIR375, MIR411*, and *MIR431* are downregulated in the cortex of AD patients, while *MIR199A2, MIR218-2, MIR24-2, MIR92A1,* and *MIR99A* are upregulated. 

These miRNAs-regulated processes are directly linked to AD, such as Aβ production or Tau phosphorylation. In particular, according to the literature, *MIR29B1, MIR24-2,* and *MIR218* have as target secretases, while *MIR219A1* and *MIR218-2* target Tau and its phosphorylation. *MIR431* modulates the Wnt pathway, *MIR92A1* is involved in memory, *MIR199A2* targets neuritin, while *MIR129-2* and *MIR99* are associated with Aβ plaque and NFT. *MIR375* is altered in tauopathy. *MIR29B1, MIR129-2, MIR219A1, MIR199A2, MIR92A1,* and *MIR1296* were already reported to be altered in AD patients, and our study confirmed their dysregulation in the cortex. Conversely, *MIR99A, MIR24-2, MIR375, MIR411, MIR431,* and *MIR218* have never been observed in the brain tissue analysis of AD patients before. Nevertheless, they are linked to neuronal functions and need deep investigation.

## Figures and Tables

**Table 1:** miRNAs that were differentially expressed in healthy subjects and AD patients, with fold change and *q*-value.

miRNA	Healthy Subjects Expression ± SD	AD Patients Expression ± SD	Fold Change	*q*-Value
*MIR129-2*	4553.64 ± 11,498.81	387.41 ± 385.06	−2.74	1.46 × 10^−2^
*MIR1296*	302.98 ± 602.29	77.88 ± 55.42	−2.41	2.43 × 10^−2^
*MIR199A2*	1.74 ± 1.97	7.45 ± 10.04	2.38	1.82 × 10^−2^
*MIR218-2*	4.51 ± 2.75	97.15 ± 239.28	3.46	1.82 × 10^−2^
*MIR219A1*	5571.76 ± 14,780.51	3.36 ± 7.21	−9.28	8.24 × 10^−8^
*MIR24-2*	6.50 ± 3.93	16.23 ± 21.56	1.72	3.19 × 10^−2^
*MIR29B1*	246.48 ± 301.94	17.75 ± 19.93	−2.72	8.24 × 10^−4^
*MIR375*	34.98 ± 13.42	12.94 ± 12.35	−1.26	9.35 × 10^−3^
*MIR411*	866.63 ± 671.94	442.34 ± 230.96	−1.19	1.30 × 10^−2^
*MIR431*	44.84 ± 55.42	19.07 ± 14.79	−1.92	1.61 × 10^−2^
*MIR92A1*	109.22 ± 114.82	448.56 ± 939.73	2.17	4.92 × 10^−2^
*MIR99A*	3250.67 ± 1624.41	5695.06 ± 2568.75	1.15	3.73 × 10^−3^

The column Fold Change highlights the difference between the expression level of the gene computed by log_2_, and the *q*-Value column shows the significance of the miRNAs that were differentially expressed after Benjamini–Hochberg correction (*q* < 0.05). AD: Alzheimer’s disease; SD: standard deviation.

**Table 2:** Role of miRNAs that were up- and downregulated between healthy individuals and AD patients.

miRNA	Biological Role Linked to AD	Pathway	Target
*MIR129-2*	Nerve injury, inflammatory response, Aβ, and NFT plaques	-	-
*MIR1296*	Neural response to oxidative stress	-	-
*MIR199A2*	Neurogenesis, neural migration, early brain development, autophagy	-	Neuritin, ERK
*MIR218-2*	Tau phosphorylation, mitochondrial respiratory chain	Wnt signaling pathway, non-amyloidogenic pathway	PTPα, ADAM17
*MIR219A1*	Tau phosphorylation, glutamate neurotoxicity	-	Tau, CAMK2G, TTBK1, GSK-3β
*MIR24-2*	Aβ production	-	NCSTN, MMP14
*MIR29B1*	Neuron survival	Amyloidogenic pathway	BACE1, Sp1
*MIR375*	Tauopathy	-	UBE3A
*MIR411*	Neuroprotective effects	-	FasL
*MIR431*	Aβ protection	Wnt/β-catenin signaling pathway	Krm1
*MIR92A1*	Structural plasticity	-	CPEB3, MEF2D, KCC2
*MIR99A*	NFT	-	-

Each miRNA was associated with the biological role that could affect AD. Moreover, where it is known, the pathway in which the miRNA is involved and each target of the miRNA are highlighted. Otherwise, “-”.

## Supplementary Materials

The following are available online at https://www.mdpi.com/2073-4425/11/9/983/s1. Table S1: Genes belonging to AD pathway in KEGG that are targets of the up- and downregulated miRNAs.
