# Supplementary material for: MicroRNAs Modulate the Pathogenesis of Alzheimer’s Disease: An In Silico Analysis in the Human Brain
Source: Genes (Basel). 2020 Aug 24;11(9):983. doi: 10.3390/genes11090983 (PMC7564652; doi:10.3390/genes11090983)
Supplement: Supplementary file 1 [file genes-11-00983-s001.zip › Table S1.docx]

**Table S1.** Genes belonging to AD pathway in KEGG that are targets of the up- and down-regulated miRNAs.

| ***MIR129-2*** | *ADAM17, AKT3, APBB1, APC, ARAF, ATG2A, ATP2A2, ATP5F1B, ATP5MC1, ATP5PD, ATP5PF, AXIN1, BACE1, BACE2, BECN1, BID, BRAF, CACNA1C, CACNA1D, CALM1, CAPN2, CASP3, CASP7, CASP8, CHRM3, CHRM5, COX4I2, COX6A2, COX6B1, COX6C, COX7A2L, COX7C, COX8A, CSF1, CSNK1E, CSNK2A1, CSNK2A2, CTNNB1, CYCS, DDIT3, DKK1, DVL1, DVL2, EIF2AK2, ERN1, FAS, FRAT2, FZD3, FZD4, FZD5, FZD6, GNAQ, GRIN2A, GSK3B, IKBKB, IL1A, IL1B, IRS1, ITPR1, ITPR3, KIF5B, LRP6, MAP2K1, MAP2K2, MAP3K5, MAPK1, MAPK10, MCU, MME, NDUFA4, NDUFA9, NDUFB3, NDUFS3, NDUFS6, NDUFV2, NDUFV3, NOX4, NRAS, PIK3C3, PIK3CA, PIK3CD, PIK3R1, PIK3R3, PLCB1, PLCB4, PPID, PSEN2, PSENEN, PSMA4, PSMA5, PSMB7, PSMC2, PSMC3, PSMD11, PTGS2, RB1CC1, RTN4, SDHA, SDHC, SLC25A5, SLC25A6, TNFRSF1A, TUBA1A, TUBA1B, TUBA1C, TUBA4A, TUBB, TUBB2A, TUBB2B, TUBB3, TUBB4B, TUBB6, ULK1, UQCRB, UQCRQ, WNT2B, WNT4, WNT5A, WNT5B, WNT7A, WNT9A* |
| --- | --- |
| ***MIR1296*** | *AKT1, AKT2, CYCS, KIF5B, KIF5C, NDUFS5, TUBA1B, UQCRC1* |
| ***MIR199A2*** | *ADAM10, AKT1, APOE, APP, ATF6, ATG13, ATP5F1B, BECN1, CALM2, CALML4, CAPN2, CDK5R1, CSNK1A1, CSNK2A1, ERN1, FAS, FZD10, FZD4, FZD6, GSK3B, IKBKB, INSR, IRS4, KIF5B, KRAS, MAPK1, MAPK8, MAPK9, MTOR, NDUFA12, NDUFA13, NDUFA2, NDUFC2, NDUFS2, NFKB1, PIK3CB, PIK3CD, PIK3R1, PSMA6, PSMD6, PSMD9, PTGS2, RELA, UQCRC2, WIPI2, WNT2* |
| ***MIR218-2*** | *ADAM17, AKT3, APC, ATP5F1B, AXIN1, CACNA1C, CALM1, COX6A1, COX7A2L, CSNK2A1, DKK2, GAPDH, GRIN2B, IKBKB, KIF5B, KIF5C, MAP2K7, MAP3K5, MTOR, NDUFA4, NDUFA6, NDUFS1, NRAS, PIK3R1, PLCB1, PPP3R1, PSMB5, PSMD12, RB1CC1, RTN4, SLC25A6, TUBB, ULK2, WNT5A* |
| ***MIR219A1*** | *AMBRA1, APC, CALM2, CHRM1, CHRM3, COX4I1, CSNK1E, CTNNB1, FZD3, IKBKB, MAPK10, NDUFAB1, NDUFB5, PPP3R1, PSMC5, PSMD6, RELA* |
| ***MIR24-2*** | *ADAM17, ADRM1, AKT3, AMBRA1, APAF1, APC, APH1A, ATP2A2, ATP5F1A, ATP5F1B, ATP5F1D, CALM1, CAPN1, CASP3, COX6B1, CSNK2A2, CTNNB1, DVL3, FADD, FZD1, FZD4, FZD5, GRIN2B, GSK3B, IL1A, IL1B, IRS1, IRS4, KIF5B, KLC2, MAP2K2, MAP2K7, NAE1, NCSTN, NDUFA2, NDUFA7, NDUFV2, PIK3CB, PIK3CD, PIK3R3, PPP3R1, PSMA7, PSMD1, PSMD11, RAF1, RELA, RTN4, TNF, TUBA1B, TUBA1C, TUBB, ULK2, VDAC1, WNT4* |
| ***MIR29B1*** | *AKT1, AKT2, AKT3, APC, ATP5F1A, ATP5F1C, ATP5MC1, ATP5MC2, BACE1, CALM3, CASP3, CASP7, CASP8, CHRM1, COX6B1, COX7A2L, CTNNB1, CYCS, DVL3, EIF2S1, FRAT2, FZD5, GAPDH, GSK3B, ITPR1, KIF5B, LPL, LRP6, MAPK10, MAPK8, NDUFS2, NRAS, PIK3C3, PIK3R1, PIK3R3, PPID, PPP3CC, PSMD7, PSMD9, RB1CC1, RELA, SDHB, SLC25A5, TNFRSF1A, TUBB, TUBB2A, ULK1, VDAC1, XBP1* |
| ***MIR375*** | *APH1B, ATP5PB, CALM3, CASP3, COX7A2L, CTNNB1, EIF2AK2, EIF2S1, FZD4, FZD8, IL1B, IL6, IRS1, KIF5B, LRP5, MAPK3, NDUFB5, PIK3CA, PPIF, PSMA6, RAF1, RB1CC1* |
| ***MIR411*** | *ATP5MC1, BACE2, COX7B, CSNK1A1, CSNK1E, FZD6, GRIN2A, GSK3B, MME, NDUFV3, PIK3CB, PSMD12, UQCRB* |
| ***MIR431*** | *GAPDH, PPIF, PSMB2, RELA, TUBA1C, TUBB, TUBB4B, XBP1* |
| ***MIR92A1*** | *ADAM10, ADRM1, APH1A, APH1B, APP, ARAF, ATP2A2, ATP5F1A, ATP5F1B, ATP5PB, ATP5PF, AXIN1, BACE1, BID, BRAF, CALM3, CDK5R1, COX4I1, COX8A, CTNNB1, FADD, FAS, FRAT2, FZD6, GAPDH, GNAQ, GSK3B, HSD17B10, IKBKB, IL1A, IRS2, IRS4, ITPR1, ITPR3, KIF5B, KLC2, KRAS, LRP6, MAPK1, MAPK8, MAPK9, NDUFA5, NDUFA7, NDUFB10, NDUFS1, NDUFS5, NDUFV3, NFKB1, NRAS, PIK3CB, PIK3CD, PPIF, PPP3CB, PSMA4, PSMA7, PSMB1, PSMB6, PSMC3, PSMD11, PSMD12, PSMD14, PSMD2, PSMD3, RTN4, SDHB, SDHC, SLC25A6, TUBA1C, TUBB, TUBB2B, TUBB3, TUBB4B, ULK1, UQCRC2, UQCRFS1, VDAC2, WNT5A, XBP1* |
| ***MIR99A*** | *AKT1, ATP5F1E, ATP5MC3, CALM2, CASP3, FZD5, FZD7, FZD8, GSK3B, IL6, MTOR, NAE1, NDUFA2, NOX4, PIK3CB, PPID, PPP3CA, PSMA2, PSMD1, PSMD12, SDHC, UQCR10, WNT2* |
